# Earthquake preparedness in higher education: institutional resilience, risk perception, and psychosocial mechanisms among academic staff

**DOI:** 10.3389/fpsyg.2026.1908089

**Published:** 2026-09-29

**Authors:** Mihaela Luminiţa Sandu, Mariana Floricica Călin

**Affiliations:** Faculty of Psychology and Educational Sciences, Ovidius University, Constanţa, Romania

**Keywords:** adaptive resilience, cognitive resilience, earthquake preparedness, higher education institutions, institutional resilience, psychological resilience, risk perception

## Abstract

**Introduction:**

Seismic hazards generate psychosocial and organizational consequences, while effective preparedness depends on the translation of institutional resources, risk perception, and resilience mechanisms into functional procedural knowledge.

**Objectives and hypotheses:**

This study tested an integrative model of earthquake preparedness among academic staff, examining institution-related perceptions, seismic risk perception, cognitive resilience, psychological resilience, adaptive resilience, preparedness engagement, and knowledge of intervention procedures. Hypotheses addressed direct associations, mediation, moderated mediation, and serial mediation.

**Methodology:**

The sample included 235 academic staff members employed in higher education institutions. Data were collected through an online multidimensional questionnaire assessing institutional, cognitive, psychological, and adaptive resilience, risk perception, preparedness engagement, and procedural knowledge. Exploratory factor analysis, internal consistency coefficients, inter-construct correlations, and multicollinearity diagnostics were used to examine the instrument's psychometric properties and the empirical overlap among the resilience dimensions. Statistical analyses included linear regression, mediation, moderated mediation, and serial mediation using the PROCESS macro for SPSS, with 5,000 bootstrap resamples.

**Results:**

Institutional resilience was significantly associated with engagement in preparedness activities. Cognitive resilience statistically explained the association between seismic risk perception and procedural knowledge, indicating that perceived risk becomes more functionally relevant when supported by cognitive processing capacities. Psychological resilience moderated this mechanism, showing that the indirect association varied according to emotional regulation capacity. Serial mediation did not support a fully sequential cognitive–adaptive pathway; instead, adaptive resilience accounted for a significant indirect association between institutional functioning and procedural knowledge.

**Conclusions:**

Earthquake preparedness in higher education emerges as an integrative psychosocial and organizational process shaped by institutional mechanisms and individual cognitive, psychological, and adaptive resources. The findings support evidence-based preparedness programs that strengthen institutional communication, cognitive processing of risk information, psychological resilience, and adaptive capacities for emergency response.

## 1 Introduction

In the context of seismic hazards, emergency preparedness is increasingly conceptualized as an integrative psychosocial and organizational process resulting from the interaction between individual psychological mechanisms and institution-related structures (Lindell and Perry, 2012; Paton, 2003). Although earthquakes generate high levels of risk perception, the literature consistently indicates that perceived risk does not automatically translate into preparedness behaviors, functioning ambivalently: it can facilitate preventive action or, on the contrary, inhibit adaptation, depending on the psychosocial resources available (Bubeck et al., 2012; Paton and Johnston, 2001; Wachinger et al., 2013).

This ambivalence reflects the fact that risk assessment is not an exclusively rational process, but one mediated by cognitive, affective and contextual factors, which influence the likelihood of engaging in preventive behaviors (Bubeck et al., 2012; Wachinger et al., 2013). Studies suggest that the transformation of risk perception into functional procedural skills depends on individuals' ability to process and integrate risk information, as well as the institutional context in which this information is provided (Grothmann and Reusswig, 2006; Lindell and Perry, 2012). In the absence of adequate cognitive and emotional resources, perceived risk may favor maladaptive reactions, such as avoidance or decision-making blockage, limiting the effectiveness of preparation (Wachinger et al., 2013).

In this context, recent literature emphasizes resilience as a central mechanism of protection and adaptation, but the functional relationships among its dimensions remain insufficiently differentiated. Existing research often treats resilience as a unitary construct or in a linear sequence, without systematically analyzing the interaction between cognitive, psychological, and institutional components in supporting disaster preparedness (Bonanno, 2004; Fletcher and Sarkar, 2013; Southwick et al., 2014).

To address this gap, the present study proposes and empirically tests an integrative conceptual model of seismic preparedness in the university environment, examining both direct associations and complex statistical mechanisms, including mediation, moderated mediation, and serial mediation.

By differentiating the functional roles of multiple forms of resilience and integrating them into a coherent theoretical architecture, the study advances understanding of disaster preparedness as a non-linear, emergent process resulting from the interaction between individual and institutional factors.

The following section reviews the relevant literature and develops the theoretical framework guiding the present study.

## 2 Literature review

### 2.1 Seismic hazards, risk perception, and psychosocial vulnerability

Earthquakes represent one of the most disruptive types of natural hazards, generating persistent material, psychological, and organizational consequences. In recent decades, the increasing frequency and impact of natural disasters have intensified global economic losses and heightened the vulnerability of critical infrastructure and exposed populations (United Nations Office for Disaster Risk Reduction, and Centre for Research on the Epidemiology of Disasters, 2020). Recent seismic events have renewed scholarly interest in how individuals assess threat and develop adaptive or maladaptive responses, with the literature documenting elevated levels of post-traumatic stress, anxiety, and depression, as well as associated impairments in cognitive and social functioning (Aksoy et al., 2025; Demirci et al., 2024; Sari et al., 2023). Recent evidence from university students exposed to the 2023 Kahramanmaraş earthquakes further indicates that earthquake-related knowledge is significantly associated with several dimensions of post-earthquake trauma, including emotional, cognitive, sleep-related, behavioral, and affective responses (Atilgan and Öztürk, 2025).

Psychological consequences of earthquake exposure may also vary according to the severity and personal relevance of disaster experiences. Among children exposed to the 2023 Kahramanmaraş earthquake, emotional stress was positively associated with behavioral problems, while bereavement, injury, hospitalization, and home damage were significant correlates of distress (Kurt et al., 2024).

Beyond individually reported symptoms, post-disaster distress may also be expressed at the collective level. A qualitative analysis of social-media discourse following the Kahramanmaraş earthquake identified recurring themes of post-traumatic stress, fear, helplessness, anger, sleep-related distress, collective trauma, and calls for psychological support, illustrating the broader psychosocial impact of major seismic events (Kaya Kiliç et al., 2025).

Beyond the immediate impact, disasters influence societal development trajectories, and post-seismic recovery requires the integration of environmental, social and economic sustainability (Collins, 2018). In this context, the assessment of seismic vulnerability and risk perception becomes essential for disaster management, with discrepancies between institutional assessments and population perceptions constituting a major barrier to the implementation of risk reduction policies (Collins, 2019).

Seismic risk analysis involves a multidisciplinary approach, combining objective assessments based on structural characteristics, seismic response models, and population exposure estimates (Ferreira et al., 2020; Gavarini, 2001; Zhai et al., 2019) with subjective dimensions associated with social perceptions and assessments of risk (Fischer et al., 2022). Risk perception is defined as a subjective assessment of the probability and severity of a disaster, integrating knowledge, expectations, and the perceived ability to cope with threatening situations (Fischer et al., 2022). At the collective level, these processes are reflected in social vulnerability, understood as the ability of communities to withstand, manage and recover from the impact of disasters (Armaş, 2008).

Seismic risk perception functions as a central cognitive mechanism, integrating previous experiences, emotional reactions, and threat anticipations (Alfuqaha et al., 2023; Sari et al., 2023).

The literature highlights its ambivalent character: intense fear can inhibit adaptation through avoidance and cognitive blocking, while moderate levels can stimulate information seeking and preparation behaviors. Negative emotions, especially fear, are negatively associated with emotional regulation and cognitive functioning, positioning risk perception at the intersection of trauma and preparation.

Despite academic and institutional advances, societal vulnerability to disasters remains high, suggesting structural limits to current approaches (Weichselgartner and Pigeon, 2015). A major explanatory factor lies in the way risk perception is conceptualized, with the literature distinguishing between the psychometric paradigm—focused on heuristics and individual judgments of risk (Fischhoff et al., 1978)—and the cultural paradigm, which emphasizes the role of collective norms and values in constructing risk (Raaijmakers et al., 2008). Overall, the perception of seismic risk is shaped by catastrophic potential, uncertainty, proximity, and recurrence (Saito, 2022), as well as the capacity of institutions to communicate technical information in an accessible and cognitively relevant manner (Savadori et al., 2022).

### 2.2 Cognitive and psychological resilience in post-seismic adaptation

Cognitive resilience refers to the capacity to maintain flexible and effective information processing under stressful conditions and to integrate risk-related information into adaptive responses. In disaster contexts, it supports the interpretation of threat information, appraisal of available resources, and planning of protective actions (Höfler, 2014). These processes are closely related to risk perception, self-efficacy, outcome expectations, and the cognitive evaluation of hazard information, all of which are relevant to preparedness behavior (Brondi et al., 2021). Evidence from earthquake-exposed populations further suggests that adaptive coping and cognitive resources are associated with more functional responses to disaster-related stress, whereas insufficient cognitive resources may be accompanied by avoidance or difficulties in translating perceived risk into preparedness actions (Aksoy et al., 2025; Demirci et al., 2024; Sari et al., 2023).

Psychological resilience complements these cognitive processes by supporting emotional regulation and affective stability under conditions of threat. Earthquake-related fear may facilitate preventive behavior when individuals possess adequate coping resources, but may become maladaptive when perceived demands exceed available psychological resources (Usta et al., 2024). Resource loss has also been associated with post-traumatic symptoms and avoidant coping following earthquakes (Löw et al., 2023). Recent cross-sectional evidence among teachers further highlights the relevance of psychological resilience in relation to post-earthquake trauma in professional populations exposed to disaster-related stress (Uzun and Atilgan, 2026).

Post-seismic adaptation is heterogeneous and depends on the interaction of individual and social resources. Resilience may coexist with psychological symptoms, while self-efficacy, cognitive reappraisal, social support, and relational resources are associated with more adaptive trajectories following stressful or traumatic events (Wang et al., 2021; Zeng et al., 2021; Zhou et al., 2019). Evidence from survivors of the 2023 Türkiye earthquakes similarly indicates that resilience, self-efficacy, and social support are relevant to psychological adaptation and recovery (İme and Dilmaç, 2025; İme et al., 2025).

Overall, cognitive and psychological resilience can be understood as complementary components of seismic preparedness. Cognitive resilience is primarily relevant to the processing, interpretation, and use of risk information, whereas psychological resilience supports the emotional regulation required to maintain effective cognitive functioning under stress. Their interplay provides a theoretical basis for examining why risk perception and institutional information may be associated with preparedness outcomes differently across individuals (Bonanno, 2004; Fletcher and Sarkar, 2013; Masten, 2021).

### 2.3 Institutional and adaptive resilience in disaster preparedness

Beyond individual resources, post-seismic adaptation is deeply conditioned by institutional and infrastructural capacities. Institutional resilience reflects the ability of organizations to implement standardized procedures, ensure effective communication, and provide logistical and psychosocial support, reducing uncertainty and psychological distress and facilitating preparedness behaviors (Diotaiuti et al., 2021; James et al., 2020). Research on disaster-risk communication indicates that the provision of official information alone does not necessarily translate into preparedness behavior; message effectiveness also depends on how risk information is framed, interpreted, and integrated into individual risk appraisal and protective decision-making processes (Lindell and Perry, 2012; Savadori et al., 2022). These findings support the idea that institutional information functions as a necessary, but not sufficient, contextual factor for activating cognitive and behavioral mechanisms involved in preparing for risk situations (Grothmann and Reusswig, 2006; Lindell and Perry, 2012).

At the organizational level, earthquake resilience includes preparedness, response capacity, and the ability to maintain or restore functionality after seismic events. At the built-environment level, seismic resilience also depends on structural vulnerability and the capacity of buildings and infrastructures to withstand and recover from earthquake-related disruption (Aroquipa and Hurtado, 2022).

Organizational preparedness is also shaped by leadership processes during crises. Recent evidence from the 2023 Kahramanmaraş earthquake context has specifically examined authentic leadership as an organizational resource relevant to crisis management in both earthquake-affected and unaffected provinces (Atilgan et al., 2025).

Crisis-management research outside the disaster-specific literature also indicates associations among crisis management, decision-making, and self-confidence in managerial contexts, supporting the broader relevance of individual decision-related resources under crisis conditions (Atilgan and Kaplan, 2022).

At the societal level, although disaster response capacity has progressively improved, human vulnerability has persisted, highlighting the limits of exclusively institutional or technological adaptation (Kelman, 2020). In this context, adaptive resilience aims to strengthen critical infrastructures and the capacity of systems to withstand, function, and recover from major seismic events. The literature highlights the role of resilient design and maintenance strategies, the continuity of critical infrastructures, and the capacity for rapid post-disaster response (Urlainis and Shohet, 2023), as well as the influence of regional and environmental factors on the development of adaptive strategies.

At the macro-functional level, adaptive resilience is conceptualized as the emergent outcome of the interaction between individual cognitive and emotional mechanisms, social networks, and critical infrastructures. Infrastructural failures can generate cascading effects that amplify social vulnerability (Tang et al., 2023). Longitudinal studies highlight the heterogeneity of post-traumatic trajectories and the role of social support, housing conditions, and cultural context in post-disaster adaptation (Goodwin et al., 2020; Sakuma et al., 2020; Yamaguchi, 2025).

## 3 Theoretical framework and hypotheses

In the context of seismic hazards, the adaptation and preparedness of affected populations result from the interaction between individual psychological processes, cognitive mechanisms, institutional structures and socio-infrastructural conditions. The proposed theoretical model is theoretically derived from established frameworks in disaster psychology and risk behavior, which emphasize that human responses to earthquakes cannot be adequately explained through a unidimensional approach. Instead, a systemic perspective is required, in which the individual and collective levels of functioning dynamically interact and mutually influence one another. Within this framework, seismic preparedness is conceptualized as the result of interdependent processes operating across multiple levels of analysis. Accordingly, the model integrates four central dimensions—psychological resilience, cognitive resilience, institutional resilience, and adaptive resilience—conceptualized as distinct yet interconnected mechanisms that jointly contribute to the development of effective preparedness behaviors.

The proposed model is grounded in established theoretical frameworks from disaster psychology and risk behavior, particularly the Protection Action Decision Model (Lindell and Perry, 2012) and social-cognitive approaches to disaster preparedness (Paton, 2003).

These frameworks conceptualize preparedness as a process in which risk perception is transformed into protective action through cognitive appraisal, resource evaluation, and contextual influences. Within this perspective, institutional information serves as an external input, while individual cognitive and emotional mechanisms determine how it is processed and translated into adaptive responses.

At the individual level, affective and physiological reactions associated with seismic risk perception influence how hazard-related information is processed and used. From a theoretical perspective, risk perception serves as an initial appraisal stage in disaster response models, activating cognitive processing and motivating information seeking (Grothmann and Reusswig, 2006; Lindell and Perry, 2012).

Within this framework, individuals first evaluate the severity and probability of a threat and subsequently engage cognitive processes that enable information integration and action planning. However, its effects are not uniformly adaptive, as excessive perceived risk may lead to cognitive overload or avoidance. Therefore, its influence on preparedness is expected to operate indirectly, through cognitive mechanisms that enable the structured processing and integration of risk-related information.

This directional assumption is consistent with established disaster response models, which conceptualize risk perception as an antecedent of cognitive processing and behavioral adaptation, rather than as an outcome of these processes.

In this dynamic, cognitive resilience is positioned as a proximal mediating mechanism, facilitating the transformation of risk perception into procedural knowledge and planning capacity.

Through cognitive flexibility, adaptive reappraisal, and coherent integration of hazard information, cognitive resilience protects the individual from maladaptive reactions and supports the conversion of perceived threat into preventive action (Grothmann and Reusswig, 2006; Lindell and Perry, 2012).

Based on these theoretical and empirical considerations, cognitive resilience is expected to serve as a key mechanism linking risk perception to procedural knowledge.

Psychological resilience operates at a complementary level, being conceptualized as an emotional regulation mechanism that conditions the efficiency of cognitive processes. It reflects affective stability and the capacity for self-regulation in stressful contexts, influencing the way in which cognitive resources are activated and used (Bonanno, 2004; Southwick et al., 2014). In the model, psychological resilience is not treated as a direct mediator but as a moderator of the relationships among institutional information, risk perception, and cognitive resilience.

This moderating role is consistent with empirical findings showing that emotional regulation capacities influence how individuals process risk-related information and respond to external inputs (Bonanno, 2004; Southwick et al., 2014). Individuals with higher psychological resilience rely more on internal regulatory mechanisms, which may reduce the impact of external information, whereas individuals with lower resilience are more dependent on contextual and institutional cues in activating cognitive processing and preparedness behaviors.

At the organizational level, institutional resilience reflects the capacity of organizations to implement procedures, communication strategies, and support systems that facilitate disaster preparedness. It is theoretically expected to be associated with preparedness behaviors and to play a contextual role, shaping the relationship between risk perception and individual decisions by reducing uncertainty and providing informational and logistical resources (Diotaiuti et al., 2021; James et al., 2020).

Institutional resilience may shape the availability, clarity, and credibility of risk-related information and may therefore be associated with how individuals cognitively process and translate this information into adaptive preparedness behaviors (Lindell and Perry, 2012).

At a macro-functional level, adaptive resilience is conceptualized as the emergent outcome of the interaction between individual psychological resources, institutional capacities, and critical infrastructures. It reflects the capacity of systems and communities to absorb disruptions, maintain functionality, and facilitate the behavioral implementation of preparedness strategies, rather than the accumulation of declarative knowledge (Tang et al., 2023).

By integrating psychological, cognitive, institutional, and adaptive dimensions, the proposed framework conceptualizes seismic preparedness as an integrative process generated by the convergence of individual resources and institution-related conditions. In this study, institutional information refers to the content and accessibility of official messages regarding seismic risk, whereas institutional functioning refers to the perceived implementation of procedures, infrastructures, and operational mechanisms for emergency preparedness.

The following hypotheses were derived from the theoretical and empirical literature reviewed above and were tested using regression-based models appropriate for the single-level structure of the data:

H1. Institutional resilience is positively associated with the level of involvement in preparedness activities.
H2. The association between seismic risk perception and knowledge of intervention procedures is statistically explained by cognitive resilience.
H3. The association between institutional information and adaptive resilience is statistically explained by cognitive resilience, with this relationship varying as a function of psychological resilience.
H4. The association between institutional functioning and knowledge of intervention procedures is statistically explained through cognitive and/or adaptive resilience mechanisms, without assuming that these mechanisms necessarily operate as a fully sequential pathway.

## 4 Materials and methods

### 4.1 Participants

The study included 235 members of academic staff recruited from multiple higher education institutions. For the purposes of this study, academic staff were defined as adult professionals currently engaged in teaching and/or academic activities within a higher education institution. Eligibility criteria required participants to be employed in an academic role at the time of data collection and to provide informed consent for participation. Individuals who were not professionally affiliated with a higher education institution in an academic capacity were not eligible to participate.

Participants were recruited using non-probability sampling methods combining convenience and snowball sampling. This approach facilitated access to a relatively dispersed professional population across different higher education settings. All participants had completed higher education, and most held advanced academic qualifications, such as master's or doctoral degrees. Detailed demographic, professional, experiential, and institutional information—including gender, exact age, academic field, years of service, previous earthquake experience, geographical location, academic rank, institutional affiliation, and the exact number of participating institutions—was not systematically retained during data collection. Consequently, subgroup comparisons based on these characteristics could not be performed.

Because recruitment was conducted through convenience and snowball sampling across multiple higher education institutions, no complete sampling frame or precisely defined source population was available. Consequently, the total size of the eligible academic staff population from which the sample was drawn could not be determined, and the sample size was not calculated using a finite-population sampling formula. The final sample of 235 participants therefore represents the number of eligible respondents who completed the questionnaire during the data-collection period. Accordingly, the sample should be regarded as a non-probability analytical sample rather than as a statistically representative sample of the national academic staff population.

Although participants were recruited from multiple higher education institutions, detailed demographic and institution-level information, including gender, exact age, academic rank, years of service, and institutional affiliation, was not systematically collected. Consequently, these characteristics could not be included as covariates in the regression-based mediation and moderation models. This limits the ability to determine whether the estimated associations are independent of demographic or professional characteristics that may also be related to earthquake preparedness, risk perception, or resilience. Accordingly, the reported coefficients should be interpreted as unadjusted associations and may be subject to residual confounding and unobserved heterogeneity. In addition, because institutional identifiers were not retained during online data collection, between-institution variability could not be examined and the data could not be analyzed using a hierarchical structure.

### 4.2 Tools

Data were collected using a multidimensional questionnaire developed specifically for this research, designed to assess seismic preparedness in the university environment. The instrument was constructed to capture both institutional and behavioral dimensions of preparedness, as well as individual psychosocial mechanisms relevant to seismic risk management, in accordance with the proposed integrative conceptual framework.

The first section of the instrument assesses the level of implementation and functionality of institutional emergency preparedness measures (e.g., earthquakes, fires, floods) through dichotomous items (YES/NO). These items address the presence of formal procedures, communication systems, training activities, evacuation plans, protective equipment, and first-aid preparedness. To complement the quantitative assessment, open-ended items were included to identify the types of communication systems and facilities available at the institutional level.

The second section measures knowledge of earthquake intervention procedures using 11 dichotomous behavioral items that assess participants' familiarity with recommended actions before, during, and after a seismic event. This section reflects the procedural dimension of individual preparedness and captures the degree to which self-protective behaviors have been internalized.

The Knowledge of intervention procedures scale comprised 11 items in both the initial and final versions of the instrument. All items were dichotomously coded (0 = no, 1 = yes) and summed to obtain a total procedural-knowledge score. The theoretical score range was 0–11, and the observed range in the present sample was also 0–11. No items were removed from this scale following the exploratory psychometric analyses.

The psychosocial section consists of items rated on a five-point Likert scale (1 = never, 5 = always) and is structured into two main subscales. Earthquake risk perception captures the cognitive, affective, and evaluative components of seismic risk, while individual resilience is conceptualized as a multidimensional construct encompassing psychological, cognitive, adaptive, and institutional dimensions. These dimensions are assessed through items related to planning, access to resources, training, emotional regulation, and social support.

The Earthquake risk perception scale comprised 10 items in both the initial and final versions of the instrument. Items were rated on a five-point Likert scale ranging from 1 to 5 and summed to obtain a total risk-perception score. The theoretical score range was 10–50, whereas the observed range in the present sample was 10–45. All 10 items were retained in the final instrument.

The cognitive, psychological, adaptive, and institutional resilience subscales were each operationalized using two items rated on a five-point Likert scale (1 = never, 5 = always). For each subscale, item responses were summed, yielding a theoretical score range from 2 to 10, with higher scores indicating higher levels of the respective resilience dimension (Table 1).

**Table 1:** Composition and scoring of the resilience subscales.

Construct	Item codes	Initial items	Final items	Response format	Scoring	Theoretical range	Observed range	*M*	SD	Items removed
Cognitive resilience	RC1, RC2	2	2	5-point Likert (1–5)	Sum score	2–10	2–8	4.94	1.54	None
Psychological resilience	RP1, RP2	2	2	5-point Likert (1–5)	Sum score	2–10	2–8	5.63	1.49	None
Adaptive resilience	RA1, RA2	2	2	5-point Likert (1–5)	Sum score	2–10	2–10	5.62	1.87	None
Institutional resilience	RI1, RI2	2	2	5-point Likert (1–5)	Sum score	2–10	2–10	5.32	1.76	None

The resilience subscales comprised the following items. **Cognitive resilience:** RC1, “I participated in information or training activities regarding appropriate behavior during an earthquake”; RC2, “I identified risks in the spaces where I carry out my activities (e.g., furniture objects) and took measures to reduce them.” **Psychological resilience:** RP1, “I am able to regain my emotional balance within a reasonable period after a crisis situation, such as an earthquake”; RP2, “I can remain calm and provide support to those around me during an earthquake.” **Adaptive resilience:** RA1, “I have a personal or institutional action plan for an earthquake”; RA2, “I have sufficient basic resources (water, food, medication, and a three-day survival kit) for at least three days following an earthquake.” **Institutional resilience:** RI1, “My workplace complies with earthquake safety standards”; RI2, “I have been involved in community activities or initiatives organized by authorities for earthquake preparedness (exercises, simulations, volunteering).” These items were rated on a five-point frequency scale ranging from 1 = never to 5 = always. The two items within each subscale were summed to create the corresponding composite score.

All items included in the initial version of the instrument were retained in the final analyses. Item retention decisions were based on both statistical and theoretical criteria. Specifically, factor loadings obtained through exploratory factor analysis were evaluated against commonly accepted thresholds, with values of 0.40 or higher considered indicative of adequate item–factor association. Items with loadings slightly below this threshold were retained if they demonstrated strong conceptual relevance to the underlying construct and contributed positively to the scale's internal consistency.

In addition, items were evaluated in terms of their contribution to scale reliability (Cronbach's α) and conceptual coherence within each factor. No items were removed from the exploratory instrument. Although a small number of indicators showed psychometric values below conventional thresholds, they were retained because of their conceptual relevance to the intended content domains and the exploratory nature of the instrument. Accordingly, the resulting factor structure should be interpreted as preliminary and requiring further independent psychometric validation. The final version of the instrument therefore remained identical to the initial item pool, ensuring transparency in the analytical process and preserving the theoretical coverage of the targeted constructs.

Overall, the instrument provides an integrated assessment of seismic preparedness in the university environment by combining institutional, behavioral, and psychosocial indicators, offering a coherent empirical basis for testing the relationships proposed in the integrative conceptual model.

### 4.3 Procedure

Data were collected online through a self-administered questionnaire distributed to academic staff using convenience and snowball sampling. Participation was voluntary, and respondents completed the questionnaire anonymously. Before completing the survey, participants were informed about the study's academic purpose, the voluntary nature of participation, and the confidentiality of their responses. The online format was selected to facilitate access to a geographically dispersed professional population and to facilitate participation among academic staff from different institutional contexts.

### 4.4 Data analysis

Statistical analyses were performed using IBM SPSS Statistics (version 31) and the PROCESS macro (version 4.2; Hayes, 2022) to test mediation, moderated mediation, and serial mediation models. To investigate the latent structure of the earthquake preparedness instrument, an exploratory factor analysis (EFA) was conducted separately for each of the four component scales: Knowledge of intervention procedures (earthquakes), Risk perception associated with earthquakes, Individual resilience to earthquakes, and Implementation & communication preparedness (Table 2).

**Table 2:** Exploratory factor analysis, parallel analysis, and internal consistency of the earthquake preparedness instrument.

Indicator	Knowledge of intervention procedures	Risk perception associated with earthquakes	Individual resilience to earthquakes	Implementation & communication preparedness
KMO	0.826	0.846	0.631	0.676
Bartlett's test	χ^2^_(55)_ = 921.823, *p* < 0.001	χ^2^_(45)_ = 1,452.843, *p* < 0.001	χ^2^_(28)_ = 540.769, *p* < 0.001	χ^2^_(78)_ = 1,160.707, *p* < 0.001
Factors suggested by eigenvalue > 1	3	2	3	4
Factors retained by parallel analysis	1	2	2	4
Observed eigenvalues retained	4.487	5.355; 1.420	2.754; 1.620	4.115; 1.577; 1.431; 1.289
Initial cumulative variance associated with retained eigenvalues (%)	40.793	67.757	54.676	64.713
Extraction	PAF	PAF	PAF	PAF
Rotation	None	Promax	Promax attempted	Promax attempted
Primary factor loadings	0.299–0.771	F1:0.589–0.940; F2:0.715–0.758	Not interpreted; inadmissible solution	Not interpreted; inadmissible solution
Factor correlation	—	0.584	Not interpreted	Not interpreted
Internal consistency (Cronbach's α)	0.846	0.890	0.702	0.747

PAF, principal axis factoring. Parallel analysis was used as the primary criterion for factor retention, supplemented by scree-plot inspection and theoretical interpretability. For the eight resilience items, parallel analysis suggested a two-factor solution; however, the corresponding PAF solution produced a Heywood case (communality > 1.0), even after increasing the maximum number of extraction iterations to 100. Consequently, factor loadings and factor correlations from this solution were not interpreted as evidence of an admissible latent structure. For the Implementation & communication preparedness items, parallel analysis supported four factors; however, the corresponding four-factor PAF solution also produced a Heywood case (communality > 1.0) despite increasing the maximum number of iterations to 100. Factor loadings and factor correlations from this solution were therefore not interpreted. Cronbach's α values refer to the complete item sets analyzed for each scale.

Missing data were minimal and handled using listwise deletion, so only complete cases were included in the analyses. This approach is considered appropriate when the proportion of missing values is low and does not substantially affect statistical power or parameter estimation. Given the absence of a systematic pattern of missingness, the data were assumed to be missing completely at random (MCAR), supporting the validity of this procedure (Little and Rubin, 2002).

To identify latent psychological constructs, the Principal Axis Factoring (PAF) extraction method was used, which models the common variance among items and is well-suited to exploring latent structures in psychological research. Given the conceptually intercorrelated nature of the investigated psychosocial dimensions, Promax oblique rotation was applied, allowing for factor correlation and providing a more realistic representation of the relationships between the theoretical dimensions.

The adequacy of the data for factorization was assessed using the Kaiser–Meyer–Olkin (KMO) index and Bartlett's test of sphericity. According to the usual methodological criteria, KMO values ≥ 0.60 were considered indicative of satisfactory sample adequacy, and the statistical significance of Bartlett's test (*p* < 0.001) was interpreted as evidence of sufficient item intercorrelations to apply factor analysis.

The number of factors to retain was determined primarily through parallel analysis, supplemented by inspection of the scree plot and theoretical interpretability. Parallel analysis was introduced because reliance on the Kaiser criterion (eigenvalues > 1) alone may lead to factor over-extraction. Principal axis factoring (PAF) was used for factor extraction. When more than one factor was retained, Promax oblique rotation was applied because the underlying dimensions were expected to be correlated. Item-level pattern coefficients were examined to determine primary factor membership and identify potential cross-loadings. Loadings of approximately 0.40 or higher were considered substantively meaningful, although coefficients slightly below this threshold were retained when theoretically interpretable and when removal was not supported by the overall psychometric pattern. For each scale, the number of factors retained, the variance explained by the retained solution, item-level factor coefficients, communalities where applicable, and internal consistency indices were reported. Cronbach's α values ≥ 0.70 were considered indicative of satisfactory internal reliability.

The revised exploratory factor analyses provided a more conservative assessment of dimensionality than reliance on the eigenvalue-greater-than-one criterion alone. For Knowledge of intervention procedures, the Kaiser criterion initially suggested three factors (eigenvalues = 4.487, 1.269, and 1.124). However, parallel analysis supported retention of a single factor, and the scree plot showed a pronounced decline after the first eigenvalue. The scale was therefore treated as essentially unidimensional, suggesting that the procedural items primarily reflect a common dimension of earthquake intervention knowledge rather than three distinct subdomains.

For Risk perception associated with earthquakes, parallel analysis supported a two-factor solution. Principal axis factoring with Promax rotation indicated that the first factor comprised items reflecting personal vulnerability, anticipated consequences, concern, and perceived inability to avoid serious earthquake-related consequences. This factor was therefore labeled “Perceived personal vulnerability and anticipated consequences.” The second factor comprised items referring primarily to perceived local earthquake exposure and anticipated environmental or infrastructural impact and was labeled “Perceived earthquake exposure and environmental risk.” Primary pattern coefficients ranged from 0.589 to 0.940 for the first factor and from 0.715 to 0.758 for the second factor. The two factors were moderately correlated (*r* = 0.584), supporting the use of an oblique rotation and indicating related but empirically distinguishable aspects of earthquake risk perception.

Inspection of the item-level Pattern Matrix did not reveal cross-loadings of sufficient magnitude to preclude interpretation of the two-factor risk-perception solution. Although several items showed secondary coefficients, their primary coefficients were substantially stronger and conceptually consistent with their assigned factor. For the admissible factor solutions, full item-level coefficients, including primary loadings and relevant secondary loadings, are reported in Supplementary Table S1 to permit direct evaluation of factor membership and potential cross-loadings. Item-level coefficients from inadmissible solutions were not interpreted or reported as evidence of stable factor membership.

For the eight items representing cognitive, psychological, adaptive, and institutional resilience, sampling adequacy was modest but acceptable for exploratory analysis (KMO = 0.631), and Bartlett's test of sphericity was statistically significant, χ^2^_(28)_ = 540.769, *p* < 0.001. The Kaiser criterion suggested three factors (eigenvalues = 2.754, 1.620, and 1.075), whereas parallel analysis supported retention of two factors. However, the corresponding two-factor principal axis factoring solution was inadmissible because a communality exceeded 1.0 (Heywood case), even when the maximum number of extraction iterations was increased to 100. Consequently, item-level factor coefficients from this solution were not interpreted as evidence of a stable latent structure. Most importantly, the present data did not provide empirical support for four clearly differentiated latent resilience dimensions corresponding to the theoretically specified cognitive, psychological, adaptive, and institutional subscales.

The four theoretically specified resilience dimensions—cognitive, psychological, adaptive, and institutional resilience—were therefore retained as brief operational composite scores, with each subdimension represented by two indicators, rather than as empirically confirmed latent factors. A supplementary confirmatory analysis previously explored for the brief resilience dimensions likewise did not yield an admissible latent measurement solution. Consequently, CFA-based estimates of composite reliability, average variance extracted, and Fornell–Larcker discriminant validity were not interpreted as valid confirmatory evidence. The four resilience scores were retained for the regression-based analyses because they corresponded to the theoretically prespecified operational constructs used in the hypotheses; however, their interpretation is explicitly limited to the operational and theoretical level and should not be regarded as evidence of confirmed factorial distinctiveness. Independent replication using more extensively developed multi-item measures is required before the four-dimensional latent structure can be considered established.

For the Implementation & communication preparedness scale, sampling adequacy was modest (KMO = 0.676), while Bartlett's test of sphericity was statistically significant, χ^2^_(78)_ = 1,160.707, *p* < 0.001. Both the Kaiser criterion and parallel analysis suggested retention of four factors. However, the corresponding four-factor PAF solution was inadmissible because a communality exceeded 1.0 despite increasing the maximum number of extraction iterations to 100. Consequently, factor loadings and factor correlations from this solution were not interpreted as evidence of a stable latent structure. The internal consistency of the complete item set was nevertheless acceptable for exploratory use (Cronbach's α = 0.747). These findings suggest that the scale may capture heterogeneous institutional preparedness practices rather than a clearly defined multidimensional latent structure and therefore requires further psychometric refinement and independent validation.

Because cognitive, psychological, and adaptive resilience entered jointly or sequentially in the regression-based mediation and moderation models, supplementary observed-score correlations and variance inflation factors were examined specifically for these three composite predictors to assess potential multicollinearity. Institutional resilience was not included in this diagnostic set because it was not entered jointly with all three individual-level resilience composites in the same regression equation.

The present study is grounded in an integrative conceptual framework that combines individual-level psychological mechanisms, such as risk perception, cognitive resilience, and psychological resilience, with institution-related perceptions, such as institutional resilience, institutional information, and institutional functioning. However, the empirical data were structured at a single level of analysis, with all variables measured at the individual participant level. No institution-level identifiers, departments, or organizational clustering variables were available; therefore, no Level 2 structure could be specified.

Accordingly, the analytical strategy did not involve hierarchical linear modeling or multilevel statistical modeling. Instead, regression-based models, including mediation, moderated mediation, and serial mediation, were estimated using the PROCESS macro for SPSS, which is appropriate for testing conditional associations in single-level data. Institution-related variables were treated as individual perceptions of institutional characteristics rather than aggregated organizational-level indicators.

Intraclass correlation coefficients were not computed because ICC estimation requires a hierarchical or nested data structure. Since no grouping variable was available, between-institution variance could not be estimated, and ICC values would not be meaningful or interpretable. Therefore, the term “multilevel” is avoided in the statistical sense and is used, where necessary, only to describe the conceptual integration of individual and institution-related constructs.

Given the cross-sectional design, all reported relationships are interpreted as statistical associations rather than causal effects. Terms such as “effect” are used only when referring to standard statistical terminology in mediation analysis and do not imply causal or temporal directionality. Descriptive statistics, score ranges, and internal consistency coefficients for the study variables are presented in Table 3.

**Table 3:** Descriptive statistics, score ranges, and internal consistency.

Construct	*M*	SD	Empirical range	Theoretical range	Skewness	Kurtosis	α
1. Institutional functioning	11.25	2.45	5–16	—	−0.470	−0.230	—
2. Procedural knowledge	9.36	2.38	0–11	0–11	−1.733	2.847	0.846
3. Earthquake risk perception	26.99	7.18	10–45	10–50	0.211	−0.272	0.890
4. Cognitive resilience	4.94	1.54	2–8	2–10	0.384	−0.367	0.605
5. Adaptive resilience	5.62	1.87	2–10	2–10	0.145	−0.564	0.038
6. Institutional resilience	5.32	1.76	2–10	2–10	0.081	−0.103	0.248
7. Psychological resilience	5.63	1.49	2–8	2–10	−0.173	−0.569	0.539
8. Engagement in preparedness activities	14.44	2.58	9–20	5–25	0.090	−0.599	0.556
9. Institutional information	5.08	1.78	0–7	0–7	−0.648	−0.699	0.683

*N* = 235. α = Cronbach's alpha. The theoretical range and internal-consistency coefficient for Institutional functioning are not reported because the original composite-scoring algorithm could not be reconstructed unambiguously from the retained item-level coding.

Descriptive statistics indicated that most study variables showed relatively limited departures from symmetry. Procedural knowledge exhibited the most pronounced distributional deviation, with negative skewness (−1.733) and positive kurtosis (2.847), indicating a concentration of scores toward the upper end of the scale. Internal-consistency estimates varied substantially across the measures. Procedural knowledge (α = 0.846) and earthquake risk perception (α = 0.890) showed good internal consistency, whereas institutional information showed marginal internal consistency (α = 0.683). Lower coefficients were observed for cognitive resilience (α = 0.605), psychological resilience (α = 0.539), engagement in preparedness activities (α = 0.556), institutional resilience (α = 0.248), and adaptive resilience (α = 0.038). These lower estimates, particularly for the brief two-item resilience composites, indicate that findings involving these operational scores should be interpreted cautiously and should not be regarded as evidence of well-established latent measurement properties.

Zero-order correlations among the study constructs are presented in Table 4. Several associations were substantial. Earthquake risk perception was strongly correlated with cognitive resilience (*r* = 0.816), adaptive resilience (*r* = 0.859), and institutional resilience (*r* = 0.885), while psychological resilience was strongly correlated with engagement in preparedness activities (*r* = 0.844). Institutional functioning was also strongly associated with institutional information (*r* = 0.832). These coefficients indicate considerable observed-score overlap among several operational constructs and reinforce the need for caution when interpreting their empirical distinctiveness. At the same time, the remaining correlations varied considerably in magnitude, and several associations were weak or statistically non-significant.

**Table 4:** Inter-construct correlations.

Construct	1	2	3	4	5	6	7	8	9
1. Institutional functioning	—								
2. Procedural knowledge	0.239^**^	—							
3. Earthquake risk perception	−0.130^*^	−0.082	—						
4. Cognitive resilience	0.052	0.141^*^	0.816^**^	—					
5. Adaptive resilience	−0.177^**^	−0.042	0.859^**^	0.618^**^	—				
6. Institutional resilience	−0.214^**^	−0.143^*^	0.885^**^	0.614^**^	0.692^**^	—			
7. Psychological resilience	−0.187^**^	−0.269^**^	0.755^**^	0.508^**^	0.609^**^	0.581^**^	—		
8. Engagement in preparedness activities	−0.151^*^	−0.116	0.741^**^	0.584^**^	0.538^**^	0.564^**^	0.844^**^	—	
9. Institutional information	0.832^**^	0.297^**^	−0.124	0.055	−0.139^*^	−0.157^*^	−0.241^**^	−0.190^**^	—

Values are Pearson correlation coefficients. *N* = 235. ^*^p < 0.05; ^**^p < 0.01.

For the regression-based models in which cognitive, psychological, and adaptive resilience were entered jointly or sequentially, variance inflation factors ranged from 1.66 to 2.00, indicating that problematic multicollinearity was not evident at the observed-score level. Nevertheless, the high zero-order correlations among several constructs, together with the limited internal consistency of some brief composite measures, mean that these diagnostics should not be interpreted as definitive evidence of discriminant validity.

## 5 Results

**Hypothesis 1**. Institutional resilience is positively associated with the level of involvement in preparedness activities.

Linear regression analysis examined the relationship between institutional resilience and engagement in preparedness activities (Table 5). The model was statistically significant, *F*_(1,233)_ = 108.96, *p* < 0.001, explaining 31.9% of the variance in engagement in preparedness activities (*R*^2^ = 0.319, adjusted *R*^2^ = 0.316).

**Table 5:** Linear regression results for the association between institutional resilience and engagement in preparedness activities.

Value indicator	Value indicator
N	235
Dependent variable	Involvement in training activities
Predictor	Institutional resilience
*R*	0.564
*R* ^2^	0.319
Adjusted *R*^2^	0.316
Standard error of estimate	2.133
ANOVA	*F*_(1,233)_ = 108.96, *p* < 0.001
Unadjusted coefficient (*B*)	0.827
SE (*B*)	0.079
Standardized coefficient (β)	0.564
*t*	10.438
*p*	<0.001
95% CI for *B*	[0.671, 0.983]
Constant (intercept)	10.038
SE intercept	0.444
95% CI intercept	[9.163, 10.912]

Institutional resilience was positively and significantly associated with engagement in preparedness activities (β = 0.564, *B* = 0.827, SE = 0.079, *t* = 10.44, *p* < 0.001). The 95% confidence interval for the unadjusted coefficient was [0.671, 0.983], indicating the stability of the estimate.

The results support Hypothesis 1, indicating a statistically significant positive association between institutional resilience and engagement in preparedness activities.

**Hypothesis 2**. The association between seismic risk perception and knowledge of intervention procedures is statistically explained by cognitive resilience.

Mediation analysis examined the role of cognitive resilience in the relationship between seismic risk perception and knowledge of intervention procedures (Table 6). Seismic risk perception was positively and significantly associated with cognitive resilience (*B* = 0.1749, SE = 0.0081, *t* = 21.52, *p* < 0.001), with the model accounting for 66.5% of the variance in cognitive resilience (*R*^2^ = 0.665).

**Table 6:** Regression estimates and bootstrapped indirect effects for the mediation of cognitive resilience in the relationship between earthquake risk perception and procedural knowledge.

Predictor/effect	Outcome	*B*	SE	*t*	*p*	95% CI (LLCI–ULCI)	*R* ^2^
Path a (X → M)							0.665
Risk perception (X)	Cognitive resilience (M)	0.1749	0.0081	21.52	<0.001	[0.159, 0.191]	
Paths b and c′(X, M → Y)							0.137
Risk perception (X)	Procedural knowledge (Y)	−0.1953	0.0349	−5.60	<0.001	[−0.264, −0.127]	
Cognitive resilience (M)	Procedural knowledge (Y)	0.9610	0.1626	5.91	<0.001	[0.641, 1.281]	
Indirect effect (X → M → Y)	Procedural knowledge (Y)	0.1681	0.0269	—	—	[0.1209, 0.2283]	Significant
Direct effect (X → Y)	Procedural knowledge (Y)	−0.1953	0.0349	−5.60	<0.001	[−0.2640, −0.1267]	Significant

In the model that simultaneously included risk perception and cognitive resilience as predictors of intervention procedure knowledge, cognitive resilience was positively and significantly associated with procedural knowledge (*B* = 0.9610, SE = 0.1626, *t* = 5.91, *p* < 0.001), while the direct effect of risk perception was negative and significant (*B* = −0.1953, SE = 0.0349, *t* = −5.60, *p* < 0.001). This model explained 13.7% of the variance in procedural knowledge (*R*^2^ = 0.137).

The indirect effect of risk perception on procedural knowledge, mediated by cognitive resilience, was significant (effect = 0.1681, BootSE = 0.0269, 95% BootCI [0.1209, 0.2283]), with the bootstrap confidence interval excluding zero.

The results support Hypothesis 2, indicating a significant mediation of cognitive resilience in the context of a direct competitive effect of risk perception. This finding indicates that risk perception is more strongly associated with procedural knowledge when accompanied by cognitive resilience, highlighting cognitive resilience as a statistical explanatory mechanism linking perceived threat to actionable knowledge.

**Hypothesis 3**. The association between institutional information and adaptive resilience is statistically explained by cognitive resilience, with this relationship varying as a function of psychological resilience.

A moderated mediation analysis was conducted using PROCESS Model 7, with bias-corrected bootstrapping (5,000 resamples), to examine whether the association between institutional information and adaptive resilience is mediated by cognitive resilience and moderated by psychological resilience (Table 7).

**Table 7:** Regression estimates and conditional indirect effects for the moderated mediation model (PROCESS model 7).

Section	Predictor/effect	Outcome	*B*	SE	*t*	*p*	95% CI (LLCI–ULCI)	*R* ^2^
A. Path a (X, W, X × W → M)	Institutional information (X)	Cognitive resilience (M)	0.6268	0.2122	2.95	0.0035	[0.209, 1.045]	0.306
	Psychological resilience (W)	Cognitive resilience (M)	0.9517	0.1783	5.34	<0.001	[0.600, 1.303]	
	X × W interaction	Cognitive resilience (M)	−0.0768	0.0342	−2.25	0.0257	[−0.144, −0.009]	
B. Paths b and c′ (M, X → Y)	Institutional information (X)	Adaptive resilience (Y)	−0.1818	0.0530	−3.43	0.0007	[−0.286, −0.077]	0.411
	Cognitive resilience (M)	Adaptive resilience (Y)	0.7602	0.0611	12.43	<0.001	[0.640, 0.881]	
C. Conditional indirect effects (bootstrapped 5,000 resamples)	X → M → Y at W = 4 (low)	Adaptive resilience (Y)	0.2428	0.0537	—	—	[0.1402, 0.3516]	Significant
	X → M → Y at W = 6 (medium)	Adaptive resilience (Y)	0.1260	0.0369	—	—	[0.0566, 0.2015]	Significant
	X → M → Y at W = 7 (high)	Adaptive resilience (Y)	0.0675	0.0472	—	—	[−0.0183, 0.1659]	Not significant
D. Index of moderated mediation			**−0.0584**	0.0233	—	—	[−0.1031, −0.0106]	**Significant**

Bold values indicate statistically significant results. For regression coefficients, statistical significance is defined as *p* < 0.05; for bootstrapped indirect effects and the index of moderated mediation, significance is indicated by a 95% bootstrap confidence interval that does not include zero.

In the first stage (Path a), institutional information was positively and significantly associated with cognitive resilience (*B* = 0.6268, SE = 0.2122, *t* = 2.95, *p* = 0.0035), and psychological resilience was also positively and significantly associated with cognitive resilience (*B* = 0.9517, SE = 0.1783, *t* = 5.34, *p* < 0.001). The interaction between institutional information and psychological resilience was significant (*B* = −0.0768, SE = 0.0342, *t* = −2.25, *p* = 0.0257). The model accounted for 30.6% of the variance in cognitive resilience (*R*^2^ = 0.306).

In the second stage (Paths b and c′), cognitive resilience was a positive and significant predictor of adaptive resilience (*B* = 0.7602, SE = 0.0611, *t* = 12.43, *p* < 0.001), while institutional information had a significant negative direct effect on adaptive resilience (*B* = −0.1818, SE = 0.0530, *t* = −3.43, *p* = 0.0007). This model explained 41.1% of the variance in adaptive resilience (*R*^2^ = 0.411). The negative direct association between institutional information and adaptive resilience was unexpected and should not be interpreted as evidence that institutional information inherently reduces resilience. Rather, given the simultaneous positive pathway through cognitive resilience, the negative coefficient may reflect a conditional or suppression-like pattern within the multivariable model and is therefore interpreted cautiously.

The analysis of conditional indirect effects showed that the indirect effect of institutional information on adaptive resilience through cognitive resilience was significant at low levels of psychological resilience (W = 4; effect = 0.2428, BootSE = 0.0537, 95% BootCI [0.1402, 0.3516]) and at medium levels (W = 6; effect = 0.1260, BootSE = 0.0369, 95% BootCI [0.0566, 0.2015]), but was not significant at high levels (W = 7; effect = 0.0675, BootSE = 0.0472, 95% BootCI [−0.0183, 0.1659]).

The moderated mediation index was significant (Index = −0.0584, BootSE = 0.0233, 95% BootCI [−0.1031, −0.0106]).

The results support Hypothesis 3, demonstrating moderated mediation: the association between institutional information and adaptive resilience is statistically explained by cognitive resilience and varies across levels of psychological resilience. This pattern suggests that the association between institutional information and adaptive resilience varies according to the individual's emotional regulation capacity, with cognitive resilience serving as a conditional statistical pathway linking contextual inputs to adaptive outcomes.

**Hypothesis 4**. The association between institutional functioning and knowledge of intervention procedures is statistically explained through cognitive and adaptive resilience mechanisms.

Serial mediation analysis was performed using PROCESS Model 6, with bias-corrected bootstrap (5,000 resamples), to examine whether the association between institutional functioning and knowledge of intervention procedures is statistically explained through cognitive resilience and adaptive resilience (Tables 8–11).

**Table 8:** Regression coefficients for the association between institutional functioning (X) and cognitive resilience (M1) in the serial mediation model (PROCESS model 6, bias-corrected bootstrap 5,000).

Predictor	*B*	SE	*t*	*p*	LLCI	ULCI	*R* ^2^
Constant	4.5752	0.4742	9.65	<0.001	3.6409	5.5094	
Institutional functioning (X)	0.0325	0.0412	0.79	0.4314	−0.0487	0.1137	0.003

In the first step of the model, institutional functionality did not significantly predict cognitive resilience (*B* = 0.0325, SE = 0.0412, *t* = 0.79, *p* = 0.431), explaining a very small proportion of the variance (*R*^2^ = 0.003; Table 8).

In the next step, institutional functionality was negatively and significantly associated with adaptive resilience (*B* = −0.1596, SE = 0.0380, *t* = −4.20, *p* < 0.001), while cognitive resilience was positively and strongly associated with adaptive resilience (*B* = 0.7617, SE = 0.0604, *t* = 12.61, *p* < 0.001). This model explained 42.5% of the variance in adaptive resilience (*R*^2^ = 0.425; Table 9). This inverse coefficient should likewise be interpreted as a conditional association estimated after accounting for cognitive resilience, rather than as evidence that institutional preparedness mechanisms directly undermine adaptive capacity.

**Table 9:** Regression coefficients for the effects of institutional functioning (X) and cognitive resilience (M1) on adaptive resilience (M2) in the serial mediation model (PROCESS model 6).

Predictor	*B*	SE	*t*	*p*	LLCI	ULCI	*R* ^2^
Constant	3.6489	0.5173	7.05	<0.001	2.6297	4.6681	
Institutional functioning (X)	−0.1596	0.0380	−4.20	<0.001	−0.2346	−0.0847	
Cognitive resilience (M1)	0.7617	0.0604	12.61	<0.001	0.6427	0.8808	0.425

In the final model, institutional functionality (*B* = 0.1965, SE = 0.0635, *t* = 3.10, *p* = 0.0022) and cognitive resilience (*B* = 0.3364, SE = 0.1261, *t* = 2.67, *p* = 0.0082) positively and significantly predicted knowledge of intervention procedures, while the effect of adaptive resilience did not reach statistical significance (*B* = −0.1797, SE = 0.1056, *t* = −1.70, *p* = 0.0902). The model explained 8.5% of the variance in procedural knowledge (*R*^2^ = 0.085; Table 10).

**Table 10:** Regression coefficients for the effects of institutional functioning (X), cognitive resilience (M1), and adaptive resilience (M2) on procedural knowledge (Y) in the serial mediation model (PROCESS model 6).

Predictor	*B*	SE	*t*	*p*	LLCI	ULCI	*R* ^2^
Constant	6.4948	0.9170	7.08	<0.001	4.6881	8.3014	
Institutional Functioning (X)	0.1965	0.0635	3.10	0.0022	0.0714	0.3216	
Cognitive resilience (M1)	0.3364	0.1261	2.67	0.0082	0.0879	0.5850	
Adaptive resilience (M2)	−0.1797	0.1056	−1.70	0.0902	−0.3878	0.0284	0.085

Bootstrap indirect effects analysis indicated a significant total indirect effect of institutional functionality on procedural knowledge (effect = 0.0352, BootSE = 0.0182, 95% BootCI [0.0057, 0.0785]; Table 11). Among the specific indirect effects, the path through cognitive resilience (X → M1 → Y) was not significant, while the path through adaptive resilience (X → M2 → Y) was significant (effect = 0.0287, BootSE = 0.0160, 95% BootCI [0.0041, 0.0685]). The full serial effect (X → M1 → M2 → Y) was not significant (effect = −0.0044, BootSE = 0.0061, 95% BootCI [−0.0222, 0.0039]).

**Table 11:** Bootstrapped indirect effects (total, specific, and serial) for the institutional functioning → cognitive resilience → adaptive resilience → procedural knowledge path (PROCESS model 6, bias-corrected bootstrap 5,000).

Indirect effect	Effect	BootSE	BootLLCI	BootULCI	Interpretation
TOTAL indirect effect	0.0352	0.0182	0.0057	0.0785	Significant
Ind1 (X → M1 → Y)	0.0109	0.0132	−0.0119	0.0423	Not significant
Ind2 (X → M2 → Y)	0.0287	0.0160	0.0041	0.0685	Significant
Ind3 (X → M1 → M2 → Y) *(serial)*	−0.0044	0.0061	−0.0222	0.0039	Not significant

Ind1, effect via cognitive resilience only; Ind2, effect via adaptive resilience only; Ind3, serial indirect effect via cognitive resilience followed by adaptive resilience.

The results partially support Hypothesis 4, indicating the existence of differential mechanisms underlying the association between institutional functioning and knowledge of intervention procedures.

These findings indicate that institutional functioning is associated with procedural knowledge through multiple, partially independent pathways rather than through a strictly sequential cognitive–adaptive process. The tested integrative earthquake preparedness model is summarized in Figure 1.

**Figure 1:**
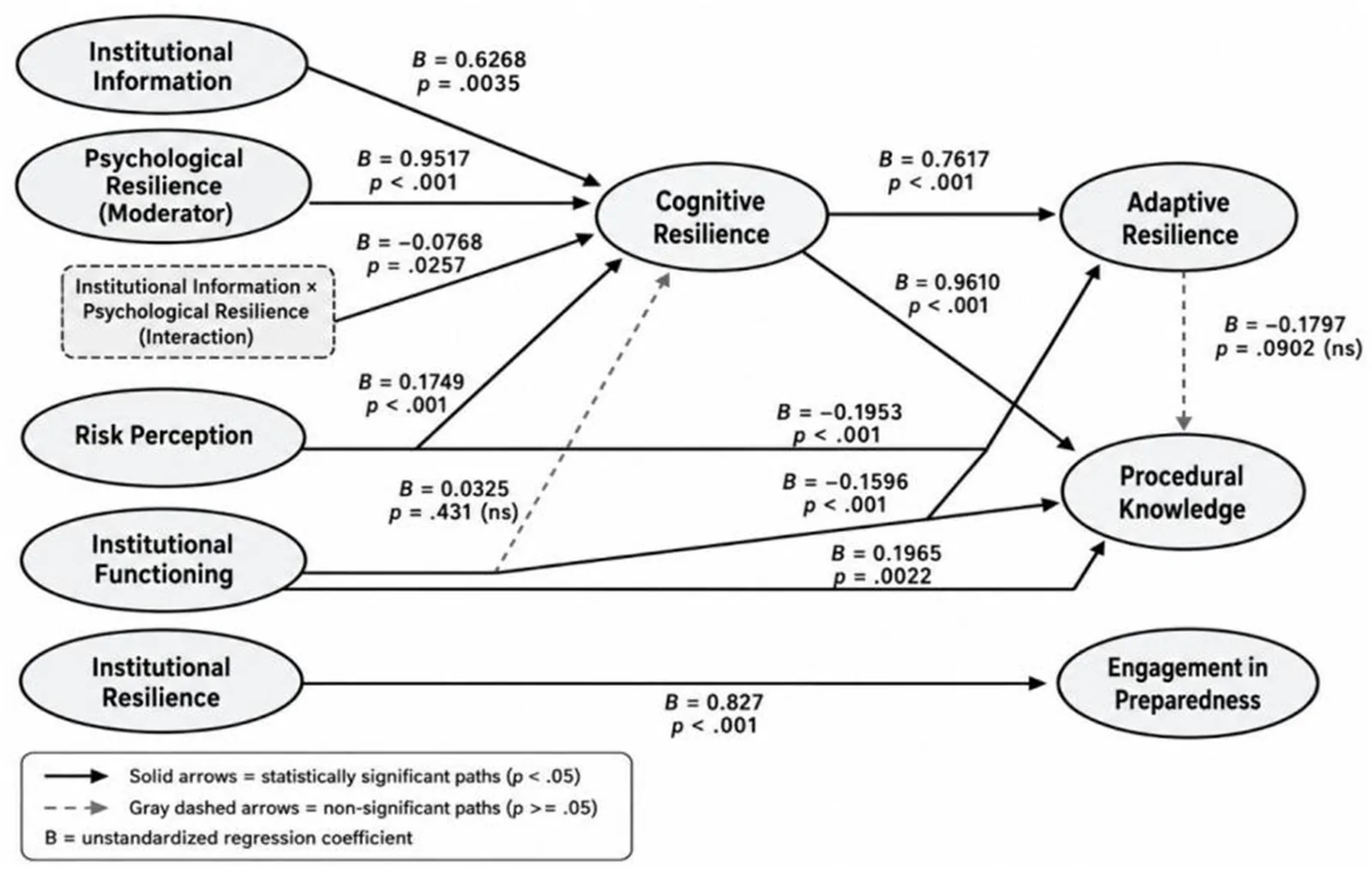
Conceptual summary of the tested integrative earthquake preparedness model. Solid arrows indicate statistically significant paths (*p* < 0.05), whereas gray dashed arrows indicate non-significant paths (*p* ≥ 0.05). Indirect and conditional indirect effects are reported in the corresponding tables and should be interpreted as combinations of component paths rather than as single direct relations.

Path coefficients represent unstandardized regression coefficients from the corresponding regression-based models. Solid arrows indicate statistically significant paths, whereas dashed arrows indicate non-significant paths. Indirect and conditional indirect effects are reported in the corresponding tables and should be interpreted as combinations of component paths rather than as single direct relations.

## 6 Discussion

The present study examined the psychological and institutional mechanisms underlying seismic preparedness in a university context by testing an integrative model that incorporates cognitive, psychological, and institutional factors, as well as adaptive resilience. Overall, the findings support systemic approaches to disaster preparedness, which conceptualize protective behavior as emerging from the interaction between individual cognitive–affective processes and contextual institutional factors, rather than from linear or unidimensional mechanisms (Lindell and Perry, 2012; Paton, 2003). This perspective is consistent with prior research emphasizing that preparedness is a dynamic and context-dependent process shaped by both internal resources and environmental inputs (Grothmann and Reusswig, 2006; Masten, 2021). Importantly, the present study contributes to this perspective by empirically examining theoretically derived associations among institution-related perceptions, cognitive and psychological resilience, adaptive resilience, and preparedness indicators within a single integrative framework.

A first key finding highlights the role of institutional resilience in relation to preparedness behaviors. Institutional capacity—reflected in procedural clarity, communication effectiveness, and access to training—was significantly associated with engagement in preparedness activities. This finding aligns with prior evidence demonstrating the importance of institutional support and risk communication in fostering preparedness (Diotaiuti et al., 2021; James et al., 2020), and extends existing research by positioning institutional resilience not only as a contextual facilitator but also as a structural component associated with behavioral engagement. Within the Protection Action Decision Model, such institutional inputs can be interpreted as environmental cues associated with protective decision-making processes (Lindell and Perry, 2012), reinforcing the relevance of organizational infrastructures for adaptive preparedness responses (Paton, 2003).

Regarding the relationship between seismic risk perception and procedural knowledge, the findings indicate that risk perception is indirectly associated with preparedness through cognitive resilience. This result is consistent with previous studies showing that risk perception may produce adaptive or maladaptive responses depending on the availability of cognitive resources (Bubeck et al., 2012; Wachinger et al., 2013). The present findings extend this literature by suggesting that cognitive resilience may represent a proximal explanatory mechanism linking perceived threat to actionable knowledge. In line with social-cognitive models, cognitive processing and information integration appear to be necessary conditions for transforming risk appraisal into adaptive responses (Grothmann and Reusswig, 2006; Lindell and Perry, 2012), thereby clarifying the mechanism through which risk perception becomes functionally relevant.

The moderated mediation results further suggest that the association between institutional information and cognitive resilience varies as a function of psychological resilience. Specifically, institutional information showed stronger associations with cognitive resilience at lower and moderate levels of psychological resilience, with diminishing effects at higher levels. This pattern is consistent with theoretical models of emotional regulation, which posit that individuals with greater psychological resilience rely more on internal regulatory capacities and are less dependent on external informational inputs (Bonanno, 2004; Southwick et al., 2014). From this perspective, psychological resilience can be conceptualized as a meta-regulatory factor associated with variation in cognitive processing, while cognitive resilience may represent one statistical pathway linking external information with adaptive outcomes (Masten, 2021). These findings are consistent with the proposed functional differentiation between cognitive and psychological resilience; however, given the exploratory psychometric status of the brief composite measures, this distinction should be interpreted at the operational and theoretical level rather than as definitive evidence of latent construct distinctiveness.

The serial mediation analysis provides further insight into the mechanisms linking institutional functioning to procedural knowledge. Contrary to traditional sequential models of resilience, which assume a linear progression from cognitive to adaptive mechanisms, the findings suggest that these processes operate through partially independent pathways, highlighting the configurational nature of disaster preparedness. This challenges dominant linear assumptions in resilience research and underscores the need to account for parallel and context-dependent mechanisms.

### 6.1 Interpreting the inverse associations between institutional factors and adaptive resilience

The inverse associations involving institutional variables and adaptive resilience warrant particular attention. Institutional information showed a significant negative direct association with adaptive resilience after cognitive resilience was included in the model, despite a positive indirect pathway through cognitive resilience. Similarly, institutional functioning was negatively associated with adaptive resilience when cognitive resilience was simultaneously considered. These coefficients should not be interpreted as indicating that institutional preparedness or information inherently diminishes adaptive capacity. Rather, they may reflect the conditional nature of institutional influences once individual cognitive resources are taken into account.

One possible interpretation is that extensive institutional information may increase the salience and complexity of seismic risk without necessarily providing individuals with sufficient opportunities to translate this information into personally meaningful adaptive strategies. Under such conditions, repeated or highly procedural information may contribute to cognitive overload, perceived complexity, or reduced personal agency, particularly when institutional communication is experienced as prescriptive rather than empowering. Disaster-preparedness models similarly suggest that information alone does not automatically produce protective action; its effectiveness depends on appraisal processes, perceived efficacy, and the individual's capacity to translate contextual cues into actionable responses (Grothmann and Reusswig, 2006; Lindell and Perry, 2012).

A complementary explanation concerns the possibility of perceived institutional substitution or a false sense of security. When preparedness responsibilities are perceived as being strongly assumed by the institution, individuals may rely more heavily on formal procedures and organizational infrastructures and invest less in personally initiated adaptive strategies. In this sense, strong perceived institutional functioning could coexist with lower self-reported adaptive mobilization without implying that institutional preparedness itself is harmful. Organizational routines may also become bureaucratically rigid when procedures are experienced as formal requirements rather than as flexible resources for individual adaptation. These interpretations remain tentative, however, because the present cross-sectional design does not permit determination of the temporal or causal processes underlying the inverse coefficients.

Importantly, the study provides preliminary evidence of functionally differentiated roles for cognitive and adaptive resilience within the tested regression-based models, although their latent psychometric distinctiveness requires further independent validation. Furthermore, the findings indicate two distinct pathways through which institutional functioning is associated with procedural knowledge: a direct pathway reflecting access to formal procedures and infrastructures, and an indirect pathway mediated by cognitive processing. This distinction aligns with theoretical approaches differentiating between knowledge acquisition and behavioral enactment in disaster preparedness (Masten, 2021; Paton, 2003). Although the coefficient linking adaptive resilience to procedural knowledge was negative (*B* = −0.1797), this association did not reach statistical significance (*p* = 0.0902) and should therefore not be interpreted as evidence of an inverse relationship. The non-significant pattern may instead indicate that adaptive resilience and procedural knowledge capture different aspects of preparedness, with adaptive resilience potentially reflecting behavioral mobilization and resource use rather than the possession of declarative or procedural knowledge. This interpretation should be tested directly in future studies using behavioral measures of emergency response.

Taken together, these findings support a non-sequential and multidimensional conceptualization of resilience, in which cognitive and adaptive components operate through complementary mechanisms.

This perspective reinforces the need for theoretical models that account for the dynamic interplay between psychological resources and institutional conditions (Lindell and Perry, 2012; Ungar, 2015). Overall, the study contributes to the literature by providing an empirically informed framework that differentiates the functional roles of cognitive, psychological, and institutional resilience within the tested statistical models, while recognizing that further psychometric validation of the resilience subdimensions is required.

## 7 Limitations

The results of the present study must be interpreted in light of a number of methodological limitations inherent in the research design and analytical strategies adopted. First, the use of a cross-sectional design limits the possibility of formulating firm causal inferences between the investigated variables. Although the relationships tested are statistically supported and are consistent with the proposed theoretical framework, the exact directionality and temporal dynamics of the identified mechanisms cannot be established with certainty based on the available data.

Second, data collection through self-report may introduce specific biases, such as social desirability, response bias, or subjective estimation errors, especially in the assessment of knowledge of intervention procedures and perceptions of institutional functioning. Although the instrument used demonstrated adequate levels of internal consistency and conceptual coherence for the study's analytical purposes, integrating objective or observational indicators (e.g., actual participation in simulation exercises, independent institutional evaluations, or procedural performance tests) could strengthen the external validity of the results. Furthermore, measuring cognitive resilience through self-report may not fully capture the contextual and dynamic variability of cognitive processes involved in real emergency situations, where time pressure and situational factors can significantly influence cognitive functioning.

Third, the sample was constituted using non-probability convenience and snowball sampling and included only academic staff from higher education institutions. Because no complete sampling frame was available and the size of the underlying eligible population could not be established, the sample cannot be considered statistically representative of the wider academic staff population. The final sample size reflected the number of eligible respondents obtained during the data-collection period rather than a probability-based sampling calculation. These characteristics limit the generalizability of the findings to other academic populations, professional groups, or national higher education systems.

An additional limitation concerns the absence of detailed demographic and professional covariates. Information on gender, exact age, academic rank, years of service, previous earthquake experience, and institutional affiliation was not systematically collected and therefore could not be incorporated into the regression, mediation, or moderated mediation models. This omission is relevant to the internal validity of the estimated associations because demographic and occupational characteristics may be associated with both the predictors and outcomes examined in the study. Accordingly, the estimated structural paths may partly reflect residual confounding or unobserved heterogeneity rather than associations that are fully independent of participant characteristics. The present findings should therefore be interpreted as unadjusted associations. Future studies should collect and statistically control for relevant demographic, professional, experiential, and institutional variables to assess the robustness of the proposed psychosocial mechanisms.

The unexpected negative conditional coefficients involving institutional information, institutional functioning, and adaptive resilience should also be interpreted cautiously. Because the analyses are cross-sectional and based on self-reported composite scores, the present data cannot determine whether these patterns reflect suppression or conditional statistical effects, differences between externally provided institutional support and personally mobilized adaptive resources, or other unmeasured mechanisms. Longitudinal and experimental studies are required to distinguish among these explanations.

Fourth, the psychometric properties of the resilience dimensions should be interpreted in light of their exploratory operationalization. Cognitive, psychological, and adaptive resilience were represented by brief two-item composite scores. Supplementary analyses showed moderate inter-construct correlations and VIF values well below conventional thresholds for problematic multicollinearity, suggesting that the composite scores were not statistically redundant at the observed-score level. However, the limited number of indicators per construct restricts the robustness of latent-variable validation. A supplementary three-factor CFA did not yield an admissible confirmatory solution, indicating that the factorial distinctiveness of the three resilience dimensions cannot be regarded as definitively established in the present sample. Consequently, the findings involving these dimensions should be interpreted as associations among operationally differentiated composite scores rather than as relationships among fully validated latent constructs. Future research should develop broader multi-item measures for each resilience dimension and test their convergent and discriminant validity through CFA in an independent sample, together with measurement invariance across relevant demographic and institutional groups.

Despite these limitations, the study provides a solid empirical basis for understanding the psychosocial mechanisms underlying seismic preparedness and offers relevant conceptual benchmarks for future investigations with more robust methodological designs and complementary analytical approaches.

Additionally, although the study integrates individual and institution-related constructs conceptually, the analyses were conducted at a single level of observation, limiting the ability to capture hierarchical, nested, or cross-level associations between institutional and individual variables. Future research should collect data with explicit institutional identifiers and apply fully specified multilevel statistical models to examine between-institution variability and cross-level associations.

## 8 Conclusions

This study provides an integrative analysis of the psychological and institution-related factors associated with seismic preparedness in the university environment, highlighting the multidimensional and non-linear nature of these processes. By testing a model integrating cognitive resilience, psychological resilience, institutional resilience, and adaptive resilience, the research clarifies how risk perception and institutional support are associated with procedural knowledge and involvement in preparedness activities. The results indicate the importance of institutional resilience in supporting seismic preparedness, highlighting the role of procedural clarity, effective communication and functional organizational infrastructures. At the same time, the findings suggest that seismic risk perception alone may not be sufficient to account for preparedness-related outcomes and that its association with preparedness is linked to cognitive mechanisms involved in processing and using relevant information.

A central contribution of the study is to highlight the differentiated functions of the resilience forms involved in seismic preparedness. Psychological resilience appears to be associated with variation in the relationship between institutional information and cognitive resilience, while cognitive resilience is associated with the integration of risk-related information and with procedural knowledge. These results support the need for flexible explanatory frameworks capable of capturing the interaction between individual resources and institutional conditions. Complementarily, the results suggest that cognitive and adaptive mechanisms contribute to seismic preparedness through partially independent routes, highlighting the non-linear and configurative nature of the processes involved.

Through this approach, the study contributes to the literature in the field of disaster psychology and risk management, proposing an understanding of seismic preparedness as an emergent process, resulting from the interaction between individual cognitive and emotional resources, institutional capacities and the organizational context. This perspective goes beyond traditional sequential models and supports an integrative conceptualization of emergency preparedness.

From an applied perspective, the results suggest that seismic risk reduction strategies in the university environment should go beyond exclusively informational approaches and include interventions aimed at strengthening cognitive resilience and adapting institutional communication to the diversity of psychological profiles. Investments in institutional infrastructure remain essential, but their effectiveness depends on their coherent integration with individual psychological resources. Overall, the study provides a coherent conceptual and empirical framework for understanding seismic preparedness as a complex, multidimensional phenomenon and offers useful benchmarks for developing policies and interventions tailored to educational contexts exposed to seismic risk.

## 9 Future research directions

Future research directions should aim to expand and deepen the mechanisms identified in the present study by using more complex methodological designs. In particular, longitudinal studies would allow examination of the temporal dynamics among risk perception, cognitive resilience, psychological resilience, and adaptive resilience, facilitating testing of the directionality and stability of the observed relationships.

Future research could also integrate experimental or quasi-experimental designs by assessing the impact of structured institutional interventions (e.g., training programs, simulation exercises, or risk communication strategies) on the activation of cognitive and adaptive mechanisms involved in seismic preparedness. Such an approach would clarify the causal role of institutional information and identify the optimal conditions for its effectiveness.

Another relevant direction is to include demographic, professional, experiential, and institutional covariates, such as gender, age, academic rank, years of service, previous disaster experience, institution type, organizational climate, and geographic exposure to seismic risk. Including these variables would allow future studies to determine whether the observed associations remain robust after statistical adjustment and to identify potential sources of heterogeneity across participant and institutional subgroups. Expanding the samples to other professional groups or to the general population would also allow testing the generalizability of the proposed model across diverse organizational and socio-cultural contexts. Finally, future research could benefit from using objective and behavioral indicators of seismic preparedness, such as actual participation in simulation exercises, performance assessments in controlled scenarios, or institutional administrative data. Integrating these measures would strengthen the external validity of the conclusions and facilitate the development of more accurate predictive models of seismic hazard adaptation.

## Data Availability

The raw data supporting the conclusions of this article will be made available by the authors, without undue reservation.
