# Supplementary material for: Earthquake preparedness in higher education: institutional resilience, risk perception, and psychosocial mechanisms among academic staff
Source: Front Psychol. 2026 Sep 29;17:1908089. doi: 10.3389/fpsyg.2026.1908089 (PMC13658207; doi:10.3389/fpsyg.2026.1908089)
Supplement: Supplementary file 1 [file Table_1.pdf]

Supplementary Table S1. Item-Level Pattern Coefficients for the Risk Perception Scale

Item	F1	F2
Feeling unsafe when hearing about earthquakes in other regions	.940	−.334
Concern about the earthquake resistance of the building	.872	—
Feeling unable to avoid serious consequences	.803	—
Feeling vulnerable to possible earthquake consequences	.727	—
Concern about effects on one’s life	.675	—
Feeling exposed to a possible major earthquake	.590	—
Authorities perceived as insufficiently prepared	.589	—
Residential area perceived as exposed to earthquake risk	—	.758
Serious anticipated impact on local infrastructure	−.234	.743
Possibility of an earthquake occurring locally	.225	.715

Note. Extraction method = Principal Axis Factoring; rotation = Promax with Kaiser normalization. Coefficients < .20 are suppressed. F1 = Perceived personal vulnerability and anticipated consequences; F2 = Perceived earthquake exposure and environmental risk.
